# Virtual environments as a novel and promising approach in (neuro)diagnosis and (neuro)therapy: a perspective on the example of autism spectrum disorder

**DOI:** 10.3389/fnins.2024.1461142

**Published:** 2025-01-15

**Authors:** Ewa Sokołowska, Beata Sokołowska, Stanisław J. Chrapusta, Dorota Sulejczak

**Affiliations:** ^1^Department of Developmental Psychology, Faculty of Social Sciences, Institute of Psychology, The John Paul II Catholic University of Lublin, Lublin, Poland; ^2^Bioinformatics Laboratory, Mossakowski Medical Research Institute, Polish Academy of Sciences, Warsaw, Poland; ^3^Department of Experimental Pharmacology, Mossakowski Medical Research Institute, Polish Academy of Sciences, Warsaw, Poland

**Keywords:** autism spectrum disorder, neurodiagnosis, neurotherapy, high ecological validity, innovative technologies, virtual environments

## Abstract

Over the last three decades, dynamically evolving research using novel technologies, including virtual environments (VEs), has presented promising solutions for neuroscience and neuropsychology. This article explores the known and potential benefits and drawbacks of employing modern technologies for diagnosing and treating developmental disorders, exemplified by autism spectrum disorder (ASD). ASD’s complex nature is ideal for illustrating the advantages and disadvantages of the digital world. While VEs’ possibilities remain under-explored, they offer enhanced diagnostics and treatment options for ASD, augmenting traditional approaches. Unlike real-world obstacles primarily rooted in social challenges and overwhelming environments, these novel technologies provide unique compensatory opportunities for ASD-related deficits. From our perspective in addition to other recent work, digital technologies should be adapted to suit the specific needs of individuals with ASD.

## Introduction

Autism spectrum disorder (ASD) is a complex neurodevelopmental disorder characterized by high heterogeneity in symptomatology and traits (Lord et al., 2018; Hodges et al., 2020; Zeidan et al., 2022; Hirota and King, 2023; de Sena Barbosa et al., 2024). It can occur in all national and socioeconomic strata, and its prevalence, estimated at 1–2%, is 4.5 times higher in men than in women (Dawson and Toth, 2015; de Lange et al., 2024). The term “autism spectrum disorder” refers to significant deficits in social communication in those affected (American Psychiatric Association, 2013; Yu et al., 2024). These individuals have difficulty adapting their behavior to social situations and—even in adulthood—may have difficulty forming close relationships and sharing emotions, behaviors or interests. In terms of profile, individuals with ASD show impairments in several domains, such as social interaction, verbal and non-verbal communication, and restricted and repetitive behaviors (Shukla and Pandey, 2020; Posar and Visconti, 2022). In terms of cognitive and social skills, persons with ASD have been shown to exhibit a wide range of variability, from high- to low-functioning autism associated with learning impairments and disabilities (Rolison et al., 2015). In most cases, ASD is associated with intellectual disability, motor coordination difficulties, attention deficits, sleep disturbances and gastrointestinal disorders (Petruzzelli et al., 2021; Hadad and Yashar, 2022; Wang et al., 2023; Zaffanello et al., 2023; McKenna et al., 2024). However, it is not uncommon for some people on the spectrum to achieve high levels of skill in visual abilities, music, art and mathematics (Simonton, 2017; Hetzroni et al., 2019; Pennisi et al., 2021).

Digital technologies have great potential for creating attractive virtual environments (VEs) that can be used for commercial, recreational, training, educational and scientific research purposes. It is noteworthy that they are also finding increasing application in many areas of human activity related to healthcare and involving many different patient groups across a wide age range, both as (neuro)diagnostic tools and as (neuro)therapeutic and/or (neuro)rehabilitative support (Bohil et al., 2011; De Witte et al., 2021; Buele and Palacios-Navarro, 2023; Khirallah Abd El Fatah et al., 2023; Cushnan et al., 2024; Ezra Tsur and Elkana, 2024). Among these groups, the most challenging is the one that includes persons with ASD (Bryant et al., 2020; Carnett et al., 2023; Bexson et al., 2024). These individuals have much to gain from using virtual technologies to better function in the real world (see in the Table 1§1). Innovative technologies make it much easier for them to master certain socially beneficial and desirable behaviors by training them in partial or complete detachment from reality, i.e., in a novel and therefore attractive way for them (see also Table 1§5–§7). While in the case of other mental disorders and deficits, there is a concern that the patient’s condition may deteriorate with contact with the VE, in the case of ASD, the patient can only improve (successfully establish and maintain new and/or enhance forms of expression and/or communication, including those used in relationships with the diagnostician(s) and/or therapist(s); Arthur et al., 2021, 2023). Not surprisingly, XR (extended reality, including virtual, augmented and mixed realities, XR:VR/AR/MR; Milgram et al., 1994; Skarbez et al., 2021) has dynamically entered the ASD diagnosis/therapy field over the past decade (Bauer et al., 2023; Alopoudi et al., 2023; Khan et al., 2024; also summarized in Table 1).

**Table 1:** Summary of advantages and disadvantages of using VEs in diagnosing and treating ASD.

Diagnosis and therapy using VEs in ASD	
Benefits	Losses and potential threats
§1. The high ecological value of digital diagnosis and therapy (Refs.: Ghanouni et al., 2019, 2020, Ghanouni and Eves, 2023; Alcañiz Raya et al., 2020a, Alcaniz Raya et al., 2020b, Alcañiz et al., 2022; Bauer et al., 2021, 2023; Dixon et al., 2020; Mouga et al., 2021; Bailey et al., 2022; Ji et al., 2022; Sosnowski et al., 2022; Abdullah et al., 2023, 2024; Adiani et al., 2024; Carnett et al., 2023; Daud et al., 2023; Kourtesis et al., 2023; Ahmadian et al., 2024; Bexson et al., 2024; Carneiro et al., 2024; Chiappini et al., 2024; Drageset et al., 2024; Ferrer et al., 2024; Gayle et al., 2024; Genova et al., 2024; Gu et al., 2024; Minissi et al., 2024; Qin et al., 2024; Rodgers et al., 2024; Simeoli et al., 2024; Toma et al., 2024; Zhao et al., 2024; Zhuang et al., 2024; Liu, 2023; Mittal et al., 2024).	§1a. Not fully reflecting reality even if diagnostic and/or therapeutic methods are ecologically accurate (Refs.: Arthur et al., 2021; Valentine et al., 2021; Mukherjee et al., 2024; Russell et al., 2024; Zhao et al., 2024; Zhuang et al., 2024).
§2. Greater diagnostic efficiency and accuracy and/or precision in assessing therapeutic effects compared to traditional methods, further supported by advanced computational algorithms that help show effects in an image-based manner and reduce time (Refs.: Alcañiz Raya et al., 2020a; Alcaniz Raya et al., 2020b; Alcañiz et al., 2022; Zhang et al., 2020; Arthur et al., 2021; Valentine et al., 2021; Carnett et al., 2023; Liu et al., 2023; Oliveira Ribas et al., 2023; Santos et al., 2023; Vacca et al., 2023; Adiani et al., 2024; Ahmadian et al., 2024; Cerasuolo et al., 2024; Corey et al., 2024; Minissi et al., 2024; Mukherjee et al., 2024; Parr et al., 2024; Pivotto et al., 2024; Qin et al., 2024; Rodgers et al., 2024; Simeoli et al., 2024; Toma et al., 2024; Wankhede et al., 2024; Zhao et al., 2024).	§2a. Occurring maladaptations to ASD, sometimes causing excessive sensory load and problems with practical functioning during diagnosis and/or therapy (uncomfortable pressure on the back of the head or distortion of the nose by the HMD, complaining about the weight of the helmet; headache, double vision or eye fatigue, discomfort, and temporary movement/balance or eye-hand coordination disorders; additionally, increased sense of isolation, etc.; Refs.: Bryant et al., 2020; Bailey et al., 2022; Adiani et al., 2024; Caruso et al., 2023; Bexson et al., 2024; Pivotto et al., 2024; Poglitsch et al., 2024; Zhao et al., 2024).
§3. The ability to personalize the novel diagnostic and/or therapeutic approaches offered—also in collaboration with people with ASD high efficiency in the early identification of problems and difficulties in ASD and/or early therapy, mainly due to the speed and relevance of the new methods (Refs.: Maskey et al., 2019b, Adiani et al., 2024; Bettencourt et al., 2024; Carneiro et al., 2024; Drageset et al., 2024; Gu et al., 2024; Liu et al., 2024; Mukherjee et al., 2024; Pivotto et al., 2024; Poglitsch et al., 2024; Qin et al., 2024; Riva et al., 2024; Rodgers et al., 2024; Toma et al., 2024; Wankhede et al., 2024; Xu et al., 2024; Zhuang et al., 2024).	§3a. Tasks do not provide the full effect of personalization, and programming and reflecting all senses, including touch and taste, is difficult. There is also a lack of collaboration with people with ASD in interaction design (Refs.: Ghanouni et al., 2019, Walsh et al., 2024; Zhao et al., 2024).
§4. Creating combined approaches—incorporating various technologies and biosignals/physiological measurements, both at the stage of diagnosis and evaluation of the impact of therapy (Refs.: Alcañiz Raya et al., 2020a; Cassani et al., 2020; Carnett et al., 2023; Oliveira Ribas et al., 2023; Minissi et al., 2024; Poglitsch et al., 2024; Simeoli et al., 2024; Zhao et al., 2024).	§4a. Difficult and still inconclusive work on establishing standards and recommendations for the use of VE technologies in ASD (Refs.: Wedyan et al., 2020; Caruso et al., 2023; Ahmadian et al., 2024).
§5. Implemented remotely, in an acceptable and friendly/home environment, and thus tame/familiar to the person with autism, diagnosing and/or monitoring the effects of therapy remotely (Refs.: Maskey et al., 2019a, 2019b; Valentine et al., 2021; Valentine et al., 2020; Lunsky et al., 2022; Gabrielli et al., 2023; Mukherjee et al., 2024; Micai et al., 2024; Russell et al., 2024; Riva et al., 2024; Wang et al., 2024; Xu et al., 2024).	§5a. Bioethical issues; not always easy to ensure the safety of digital users and their data; and the experimental nature of some interactions (they are not standard; Refs.: Alcañiz Raya et al., 2020a, Alcaniz Raya et al., 2020b, Alcañiz et al., 2022; Bryant et al., 2020).
§6. The ability to easily repeat any component of the diagnosis and/or repeat therapeutic exercises (especially movement exercises) and provide rapid and fully automated (bio)feedback (Refs.: Ghanouni et al., 2019; Dixon et al., 2020; Wedyan et al., 2020; Zhang et al., 2020; Almeida et al., 2023; Carnett et al., 2023; Vacca et al., 2023; Kourtesis et al., 2023; Corey et al., 2024; Liu et al., 2024; Xu et al., 2024; Zhao et al., 2024).	§6a. Impossibility of repetitive and prolonged work due to the particularly high burden of cybersickness in both diagnostic and therapeutic processes (Refs.: Bryant et al., 2020; Bexson et al., 2024; Zhao et al., 2024).
§7. The ability to adjust the parameters of the diagnostic and/or therapeutic work according to the sensory hypersensitivity and other altered needs of the autistic person (ease of controlling various parameters of the digital environment and increasing the sense of security), as well as increasing affordability (Refs.: Ghanouni et al., 2019; Johnston et al., 2020; Wedyan et al., 2020; Bauer et al., 2021, 2023; Carnett et al., 2023; Daud et al., 2023; Liu et al., 2023; Bennewith et al., 2024; Chiappini et al., 2024; Pivotto et al., 2024; Qin et al., 2024; Zhao et al., 2024; Zhuang et al., 2024).	§7a. Digital exclusion due to cost, as well as lack of adequate preparation for the use of new methods (very advanced technologies remain expensive for both the individual user and institutions), not every measurement can be used, it is not always convenient or intuitive to use different techniques, certain expected functions or dynamics are missing, for example (Refs.: Lorenzo et al., 2022, 2023; Ahmadian et al., 2024; Corey et al., 2024; Bettencourt et al., 2024; Chiappini et al., 2024; Pivotto et al., 2024; Toma et al., 2024; Zhao et al., 2024).
§8. Attractive and motivating forms of innovative digital technology for patients and participation in both diagnosis and therapy to a greater extent than conventional approaches due to the effect of novelty, differentiation of functions, and the wide range of functions that can be improved (Refs.: Ghanouni et al., 2019; Bryant et al., 2020; Johnston et al., 2020; De Luca et al., 2021a, 2021b; Iosa et al., 2022; Bailey et al., 2022; Abdullah et al., 2023, 2024; Almeida et al., 2023; Caruso et al., 2023; Daud et al., 2023; Gabrielli et al., 2023; Vacca et al., 2023; Ahmadian et al., 2024; Gu et al., 2024; Liu et al., 2024; Pivotto et al., 2024; Poglitsch et al., 2024; Rodgers et al., 2024; Toma et al., 2024; Walsh et al., 2024; Wang et al., 2024; Xu et al., 2024; Zhao et al., 2024; Quintar et al., 2025).	§8a. Ancillary or experimental use of new methods, uncertainty about relying solely on methods that are new and not always understood (Refs.: Dixon et al., 2020; Johnston et al., 2020; Bauer et al., 2023; Carnett et al., 2023; Daud et al., 2023; Santos et al., 2023; Gayle et al., 2024; Mukherjee et al., 2024; Riva et al., 2024; Russell et al., 2024; Zhao et al., 2024).
§9. Involving the family in a meaningful way and giving them a chance to learn about new therapeutic methods from specialists (Refs.: Ghanouni et al., 2019, Maskey et al., 2019b, Wedyan et al., 2020; Angell et al., 2024; Micai et al., 2024; Poglitsch et al., 2024).	§9a. Requiring additional persuasion and/or training not only for individuals with ASD but also for their family/caregivers and medical personnel (Refs.: Angell et al., 2024; Zhao et al., 2024).

Overall, modern technologies eliminate unnecessary stimuli, reduce the possibility of distraction, and enable people with ASD to interact in a structured and personalized way, providing opportunities to work on the way they express emotions. VEs, unlike real life, allow the use of a variety of selected (to avoid over-stimulation) contact channels. Nowadays, VE users have such a wide range of digital options and combinations to choose from that it even requires a new approach(es) to categorize them, as researchers themselves also point out (De Witte et al., 2021; Tani et al., 2024).

## The usefulness of VEs for the ecological diagnosis of ASD

Neuroscientists emphasize the high ecological value of XR tools (Forbes et al., 2016; see Table 1§1). The ecological value is understood as creating/adapting to near-natural conditions, hence diagnosis and therapy based on these techniques is referred to as ecological diagnosis and ecological therapy. In the traditional approach, ASD, like any other disorder, requires a general examination of skills and interests using an appropriate set of questions that can be incorporated into a clinical interview with an adult or caregiver to explore and identify typical autistic traits (Woods and Estes, 2023). In most cases, diagnostic assessments of individuals with ASD focus exclusively on problems without considering the patients’ strengths, which is particularly evident with ASD (Urbanowicz et al., 2019; McKernan et al., 2020; Woods and Estes, 2023). Implementing diagnostic tools targeting patient strengths is much easier with digital tools than with traditional tests. Mukherjee et al. (2024) reviewed studies evaluating a variety of digital technologies, from mobile (laptops, cell phones, smart toys) to desktop (desktop computers, virtual platforms). These can be used to demonstrate computer games or record children’s behavior and expressions. Subsequent computer analysis of children’s interactions with these technologies can effectively distinguish between autistic and non-autistic children, providing a very promising rationale for (automated) screening for autism risk. In addition, appropriately designed tasks assessing social responses and hand and body movements can be the basis for very effective differentiation between autistic and typically developing children. They will also be invaluable in monitoring their development. Exposure to VEs itself may be an opportunity to test interests that tend to become rigid and stereotyped.

To date, the results of ongoing studies involving patients with ASD show many advantages, especially in terms of both early diagnosis and accuracy (Koirala et al., 2021; Mukherjee et al., 2024), in a perspective supported by other applied methods/techniques, e.g., inclusion of electroencephalography, magnetoencephalography, tractography, etc. (Evans et al., 2017; Bosl et al., 2018; Lorenzetti et al., 2018; Li et al., 2024; Rhodes et al., 2024; Schielen et al., 2024), considering also the increasingly common computational science (Rosenberg et al., 2015; Qin et al., 2022; Noel and Angelaki, 2023), including popular machine learning algorithms (Banos et al., 2024; Shrivastava et al., 2024; Wei et al., 2024). For example, machine learning/artificial intelligence approaches allow not only early and accurate diagnosis of ASD, but also early recognition of adverse symptoms that may accompany the use of virtual tools (Table 1§2a, §6a). Moreover, the inclusion and advanced analysis of data sets of various biosignals greatly increase the accuracy and reliability of ecological digital diagnostics. Bosl and Ellen (2023) proposed a new concept for the future of neurodiagnostics as a new science of clinical neuroinformatics (for functional brain monitoring) also related to VE development. Currently, this is not common practice in this patient population, although their results are particularly promising in diagnostics that, when implemented in highly ecological VEs, already provide a basis for better ecological diagnostics compared to traditional methods used to date (see Table 1§1–§3). Equally promising are the results of integrating VEs with conventional therapies and rehabilitation programs (Table 1§4, §7, §8).

## The usefulness of VEs for the ecological therapy of ASD

Various virtual tools allow users to practice skills in realistic but well-controlled environments. VEs can offer several benefits, including predictability (so important for people who seek repetition), structure (which can be predictable, which can have a calming and/or soothing effect), customizable task complexity (which allows for unique adaptation to the difficulties of a person who may be sensitized to certain auditory or visual, olfactory or tactile stimuli). In addition, there is the issue of control, providing realism and immediate feedback on progress, which supports the effects of assessment and reinforcement (Waseem et al., 2016; Bozgeyikli et al., 2018). Therapy for people with ASD is ecological in that it allows for the development of behaviors that are later transferred to everyday life. In this respect, it is identical to traditional therapies—all the advantages and disadvantages of modern technology can be freely traced. As a result, for more than two decades there has been a steadily growing need to create and develop VE-based interventions dedicated to individuals with ASD (Aresti-Bartolome and Garcia-Zapirain, 2014). This is not surprising, given the increasing prevalence of the disorder (Zeidan et al., 2022; Talantseva et al., 2023). In this situation, each emerging new approach opens the prospect of a much-needed increase in the effectiveness of treatment for the disorder. Forman et al. (2021) pointed to improving motor learning at home in neurological patients by incorporating modern information and communications technology (ICT). The researchers examined three basic categories of training (including external personalized input from the therapist): training with sensory stimuli, training with digital information exchange, and telerehabilitation. They indicated that easy-to-apply and intelligent solutions with precise feedback and individualized training methods/suggestions are essential for home training. This home-based approach to motor neurorehabilitation was aptly described by the researchers as “neuroplasticity at home.” Rehabilitation interventions can be used to modulate adaptive neuroplasticity, reduce cognitive-motor impairment and improve daily activities in patients with brain-based difficulties, including neurodevelopmental problems. This is a promising direction also in technology-assisted (neuro)therapy for people with ASD.

Many studies, such as those presented in Table 1 and others (e.g., Ip et al., 2018; Liu et al., 2021) using VEs, demonstrate the motivational-emotional aspect of aroused interest. Digital technologies (VEs) need to be (a) tailored to and well tolerated by people with ASD, (b) flexible in use, and (c) seamlessly implemented (Ke et al., 2022; Martin et al., 2021; Table 1§7–§9). Therapy can be delivered at home and can be highly individualized and intensive (Forman et al., 2021). Global trends in the use of VEs in working with people with ASD (including caregivers) can already be observed (Nie et al., 2021; Sanku et al., 2023; see also Table 1). Overall, recent advances in ASD diagnosis and therapy are an excellent example of how innovative XR-based technologies are entering and changing the lives of many patient groups for the better.

## The main limitations of using VEs for the diagnosis and therapy of ASD

Current research points to the desirability of using VEs in individuals with ASD provided that the nature of the difficulties associated with their acquisition (rigid adherence to these techniques, excessive fascination or even preoccupation with certain technical elements, etc.) is resolved. In this regard, important findings have been presented in relevant studies using VR. It is worth noting that both IT/ICT experts, researchers, clinicians and users of XR techniques themselves additionally point to financial issues as a potential source of (a) difficulties in developing and implementing new methods, as well as (b) exclusion due to differences in wealth (regions, environments, user groups), and consequently (c) the still insufficient dissemination, popularization and accessibility of these modern techniques. Assistance and support for autistic people also depends on public awareness and knowledge. Beneficial in this regard is the use of new technologies in the field of education, better attitudes and greater openness to autism (Koniou et al., 2023).

## Discussion on the prospects of research using digital technologies for ASD

The development of digital technologies, especially those based on VEs, is setting promising new trends for clinical neuroscience, clinical neuroinformatics and modern medical practice (Emmelkamp and Meyerbröker, 2021; Essoe et al., 2022; Buele and Palacios-Navarro, 2023; Riva et al., 2024). Over the past decade, they have also entered in ASD field (Bailey et al., 2022; Chen et al., 2022; Robles et al., 2022; Carnett et al., 2023; Leharanger et al., 2023; Chung et al., 2024; Hall et al., 2024; Herrera et al., 2024; Maddalon et al., 2024), and the example of working with individuals with ASD can be used to highlight the benefits and drawbacks/risks of digital technology (Table 1). There is intense research in the field of diagnosis and rehabilitation using VEs, including important new approaches such as neurodiagnostics and neurotherapy, and ASD is not an exception, but rather an ideal representative of this trend (Bonner, 2015; Pellicano and den Houting, 2022; see Table 1). Of particular interest are the ideas put foward by Bosl and colleagues, for example, about the future of neurodiagnostics and the emergence of the new science of clinical neuroinformatics (Bosl and Ellen, 2023). This is an extremely important advance in the diagnosis and treatment of ASD, as abnormalities in neuronal connections have been linked to ASD. Electroencephalography allows the assessment of neural network architecture, providing additional information and important data incorporated into ASD diagnosis/recognition using digital technologies (Bosl, 2018; Bosl et al., 2018; Bogéa Ribeiro and da Silva Filho, 2023; Table 1§4). This makes it possible to develop new, more effective methods in this area of research.

Viruega and Gaviria (2022) clearly state that neurorehabilitation must address multiple aspects of the person through a comprehensive analysis of actual and potential cognitive, behavioral, emotional and physical skills, while increasing awareness and understanding of the treated person’s new self. Researchers emphasize that each person has own rhythm, unique life history and personality construct. This makes it imperative that all of these elements be tailored to the patient’s individual needs. This also applies to autistic people, who have many non-standard needs and require special care in their daily lives (Płatos and Pisula, 2019; Hodges et al., 2020; Wang et al., 2023; Table 1). There are many indications that innovative technologies, especially those using VEs, may offer new approaches that meet the above requirements, not only for treatment, but also for more effective (neuro)diagnosis along with (self-)monitoring (Valentine et al., 2020; Mekkawy, 2021; Table 1§3). Importantly, these technologies also allow, family members, caregivers and/or medical personnel to be more involved in the care, support and treatment of individuals with ASD than was previously possible (Kuhlthau et al., 2020; Zlomke and Jeter, 2020; Yao et al., 2024). There are also many indications that, in the future, innovative technologies may be more effective and helpful for people with ASD—mainly because they enable contact that is not fraught with “overloading”/over-stimulating, while effectively encouraging and allowing for the practice of beneficial social behaviors (Hutson, 2022). Such forward-looking designs have been proposed in various studies (Valentine et al., 2020; Mekkawy, 2021; Table 1).

Many studies and review articles address the evaluation and treatment of ASD with VEs (Miller et al., 2020; see Table 1). The benefits of using these technologies can be summarized as individualized and flexible therapy. At the same time, difficulties can be associated with the need to purchase equipment and to match programs and planned interventions to the capacity of institutions to support individuals and their families. There is also growing interest in the use of VEs not only in clinical settings, but also at home, i.e., for home VR/AR/MR training and telerehabilitation (Lin et al., 2023; also Table 1§5). The researchers rightly suggest that both of these methods can be effectively used as an extension of conventional therapy. Home conditions allow for daily exercise if equipment requirements are not too high and modifications to the application do not impose an undue financial burden on the individual or supporting organization.

This is only the beginning of the road due to the unique needs of individuals with ASD compared to other patient groups, for whom various programs/tools/systems using innovative neurotechnologies have already been developed (Nielsen et al., 2015; Massetti et al., 2018; Micera et al., 2020; Morone et al., 2023; Painter et al., 2024). Researchers indicate that VEs enable, for example, personalization of treatment using an adaptable therapeutic platform, which can improve patient participation/engagement and increase acceptance and adherence to long-term treatment programs (Mukherjee et al., 2024; Table 1§3). Most promising—and avoiding large financial burdens or difficulties of a different nature—are approaches that combine various previously used techniques. Research combining traditional methods and new techniques is ongoing (Šlosar et al., 2022; Table 1§4). A study by Gabrielli et al. (2023) shows the effectiveness of such approaches. Zanatta et al. (2023) presented a structured approach using XR in rehabilitation. In addition, therapy for persons with ASD skillfully combines pharmacotherapy with virtual and other interventions (McCracken et al., 2021; Henneberry et al., 2021; Jensen et al., 2022; Yenkoyan et al., 2024).

Regarding the use of virtual technologies in the diagnosis and treatment of ASD, it is clear that there are currently deficits in the following areas: (a) guidelines and design considerations for creators of virtual worlds, (b) general recommendations for teachers and caregivers/parents of children and adults with ASD, and (c) specialized research for neuropsychologists or neurotherapists to address the various specific needs of persons with ASD in terms of social communication, perception of stimuli (including those relevant to social interactions), and interest patterns (see Table 1§1a). The observed rapid development of digital technologies and the emerging possibilities for their application in ASD research allow the indicated gaps to be gradually filled. This is a path of promising prospects for the development of digital neuropsychology, neurodiagnostics and neurotherapy for ASD. Current findings also underscore the need for collaboration between neuroscientists, digital creators/providers, ASD users and their parents/guardians, and medical personnel to ensure that the diagnosis/care/therapy offered is reliable, accurate and of high quality. Accordingly, recommendations are also being formulated for them, especially for individuals with ASD and caregivers (Hoang et al., 2024; Table 1§9). It should be noted that recent findings are already indicating/creating new directions for ASD research in modern basic and clinical (digital) neuroscience.

The use of VEs (e.g., VE alone or in combination with other methods/technologies) can also be accompanied by unfavorable effects, such as the occurrence of side effects/cybersickness (Conner et al., 2022; Martirosov et al., 2022; Sokołowska, 2023, 2024; Table 1§2a, §6a), as well as those resulting directly from the nature of the disease itself (Hirota and King, 2023; Zhuang et al., 2024). In most of the current studies, benefits far outweighed adverse effects or potential losses/risks (Table 1).
